# Behavioral Response of Weaned Pigs during Gas Euthanasia with CO_2_, CO_2_ with Butorphanol, or Nitrous Oxide

**DOI:** 10.3390/ani10050787

**Published:** 2020-05-01

**Authors:** Enver Çavuşoğlu, Jean-Loup Rault, Richard Gates, Donald C. Lay

**Affiliations:** 1 Department of Animal Science, Faculty of Veterinary Medicine, Bursa Uludag University, 16059 Bursa, Turkey; 2 Institute of Animal Welfare Science, University of Veterinary Medicine, A-1210 Vienna, Austria; Jean-Loup.Rault@vetmeduni.ac.at; 3 Egg Industry Center, 1020 NSRIC, 1029 N University Blvd, Iowa State University, Ames, IA 50011-3611, USA; rsgates@iastate.edu; 4 USDA-ARS, Livestock Behavior Research Unit, West Lafayette, IN 47907, USA; Don.Lay@ars.usda.gov

**Keywords:** gas flow rate, sus scrofa, swine, euthanasia, welfare

## Abstract

**Simple Summary:**

Pig farmers are forced to euthanize a significant number of pigs due to injuries, hernias, or unthriftiness. The majority of pigs are euthanized using carbon dioxide gas asphyxiation. However, the humaneness of carbon dioxide is being increasingly questioned. An alternative is the use of nitrous oxide gas. We conducted this study to compare the euthanasia of young pigs using nitrous oxide or carbon dioxide. In addition, we tested the administration of a pain relief drug prior to carbon dioxide exposure to determine if we could eliminate behaviors indicative of pain. Pigs became unable to control their muscle movement, breathed heavily, and lost posture at the same time regardless of treatment. Pigs exposed to both gases showed heavy breathing and open-mouth breathing prior to losing posture. However, pigs exposed to carbon dioxide made more escape attempts but fewer squeals than pigs exposed to nitrous oxide. Administration of pain relief prior to exposure to carbon dioxide did not alter behaviors indicative of pain. The findings are inconclusive as to whether using nitrous oxide is significantly better than using carbon dioxide, but the results show that its use is just as effective, and possibly more humane.

**Abstract:**

The swine industry is often forced to euthanize pigs in the first few weeks of life due to injuries, hernias, or unthriftiness. The majority of pigs are euthanized using carbon dioxide (CO_2_) gas asphyxiation but concerns as to the humaneness of CO_2_ are increasing. This study compared the euthanasia of weaned pigs using N_2_O (N_2_O; *n* = 9) or CO_2_ (*n* = 9), at 50% and 25% min^−1^ exchange rate, respectively. In addition, we administered an analgesic prior to euthanasia with CO_2_ (CO_2_B) exposure as a third treatment (*n* = 9) to elucidate behaviors indicative of pain. Pigs in the CO_2_ and N_2_O treatments lost posture at similar times (latency of 145.0 ± 17.3 and 162.6 ± 7.0 s respectively, *p* > 0.10), while the CO_2_B treatment pigs lost posture the soonest (101.2 ± 4.7 s, *p* < 0.01). The pigs in the CO_2_B treatment made more escape attempts than the CO_2_ or N_2_O pigs (16.4 ± 4.2, 4.7 ± 1.6, 0.3 ± 0.2, respectively; *p* < 0.0004). However, pigs in N_2_O squealed more often than either the CO_2_ or CO_2_B pigs (9.0 ± 1.6, 2.8 ± 1.2, 1.3 ± 0.6, respectively, *p* < 0.001). Given the similar time to loss of posture and shorter time displaying open mouth breathing, N_2_O may cause less stress to pigs; however, the greater number of squeals performed by these pigs suggests the opposite. It was not apparent that any behavior measured was indicative of pain. In conclusion, N_2_O applied at a 50% min^−1^ flow rate can be an alternative to CO_2_ for pig euthanasia.

## 1. Introduction

Pork is the most consumed livestock meat in the world, with 1.4 billion pigs slaughtered each year [1]. Unfortunately, each year approximately 7.65 million newborn pigs in the US need to be euthanized due to various problems, including injury and starvation (calculated from production and loss data [2]). In addition, a significant number of neonatal pigs have to be euthanized prior to weaning due to hernias, lameness, and lack of thriftiness. Thus, ensuring a humane death for these animals represents a significant opportunity to safeguard animal welfare.

Any method of euthanasia should minimize pain and distress, induce rapid loss of consciousness, and a quick death [3,4]. The most common methods of euthanasia are blunt force trauma for neonatal pigs and CO_2_ for neonates and older pigs. There are aesthetic concerns with blunt force trauma, and if done incorrectly, the pig can suffer. Carbon dioxide is widely used in the swine industry with automated gas chambers designed for on-farm use, typically using a fill flow rate of 25% of the chamber volume per minute (half-life 2:46). However, CO_2_ is aversive to pigs [5,6] and is a welfare concern [7]. Research in our laboratory [6] found that pigs exposed to CO_2_ squealed and flailed when concentrations of CO_2_ reached about 20%, which was interpreted as an aversive response. The recommendation for the use of CO_2_ is largely based on the speed at which it induces unconsciousness, as well as being economically affordable, widely available, and relatively safe to use for operators, despite being aversive to pigs. Therefore, the use of CO_2_ remains a significant welfare concern for producers, scientists, and the public.

Nitrous oxide (N_2_O) is one of the most common agents used in anesthetic practice for humans [8]. It is commonly used in human dental practices and referred to as ‘laughing gas’ due to its analgesic, sedative, and anxiolytic properties [9]. In mice, N_2_O mixed with CO_2_ decreased the time to loss of consciousness compared to CO_2_ alone [10]. In pigs, Rault et al. [11] showed that N_2_O was less aversive than CO_2_ and was capable of inducing anesthesia [6]. In our previous research [12] to determine if a two-step process of using N_2_O followed by CO_2_ would be more effective than N_2_O alone, one group of pigs was exposed to N_2_O at a flow rate of 25% replacement [3] for 6 min before being exposed to CO_2_ and the other group was directly exposed to CO_2_. All pigs in the N_2_O treatment lost posture (a sign of the onset of loss of consciousness) prior to entering the CO_2_. However, because the pigs in the N_2_O group were able to spend more time upright, they also had more time to squeal and attempt to escape; therefore, it could not be concluded whether the two-step method was more humane than CO_2_. The authors speculated that increasing the flow rate of N_2_O would cause pigs to lose posture sooner and those behaviors potentially indicative of aversiveness would be reduced.

It is generally believed that behaviors, such as heavy breathing, open-mouth breathing, squeals, and escape attempts, are signs of varying degrees of distress [6,13,14]. Importantly, it would be helpful to determine if these behaviors are also indicative of pain. In order to value one method of euthanasia over another, both pain and distress need to be assessed and alleviated. For instance, pigs exposed to CO_2_ lose posture quicker than those exposed to N_2_O, using similar flow rates for each gas [11]. However, if the pigs that were exposed to CO_2_ experience pain and distress before losing posture and those exposed to N_2_O do not experience pain or distress, then N_2_O would be considered a more humane method.

Therefore, we sought to determine if increasing the flow rate for N_2_O would decrease the time to loss of posture to be equivalent to CO_2._ Further, we conducted the following study to determine if behaviors indicative of pain could be elucidated by using an analgesic prior to euthanizing pigs with CO_2_. We hypothesized that: (1) A greater flow rate of N_2_O would decrease the latency to loss of posture and therefore also reduce squeals and escape attempts, and (2) pigs administered butorphanol and exposed to CO_2_ would squeal less and perform fewer escape attempts. Because both treatments deprive the pig of oxygen, we expected that heavy breathing, open-mouth breathing, and gaping to occur in both treatments.

## 2. Materials and Methods

### 2.1. Animals and Housing

All research was approved by the Purdue University Animal Care and Use Committee (#1801001687). The pigs were the progeny of a commercial crossbred line born to sows from a local producer. The research was conducted at the local farm. Weaning aged pigs, approximately 21 days of age, which were destined to be euthanized, were used for the project. This farm produced weaned pigs for sale; thus, any pig that was deemed not to meet the quality standards for sale had to be euthanized. Reasons for euthanasia included: Injuries, prolapse, hernia, lame, and lightweight. The majority (72%) were males who exhibited scrotal hernias. For each treatment, 2 pigs were euthanized together in 9 repetitions; thus, 54 pigs in total were euthanized (2 pigs × 9 repetitions × 3 treatments).

The euthanasia gas chamber was the same as that used in our previous research [12]. Briefly, the chamber was 61 × 38 × 46 cm (Euthanex^®^ Ag Pro^TM^, NutriQuest Inc., Mason City, IA, USA) that was modified with acrylic viewing windows to the front and back sides. Battery-operated lights were secured to the top interior of the box to provide more visibility for video recording. Gas was delivered to one side of the box after passing through a mass flow controller (GFC47, Aalborg Instruments & Controls, Inc., Orangeburg, NY, USA). Gas exited the tank through ducting connected to an exhaust fan (FR100, Fantech, Lenexa, KS, USA), which kept the chamber under negative pressure as confirmed via a manometer and served to flush the chamber between treatments. Gas was delivered at a 25% replacement rate per min for the CO_2_ treatments, as recommended per the American Veterinary Medical Association (AVMA) [3], and at a 50% replacement rate for the N_2_O treatment. The effect on the O_2_ concentration at these replacement rates meant that O_2_ was depleted by half (from 20% to 10%) after 2.77 and 1.39 min for the 25% and 50% replacement rates, respectively.

### 2.2. Procedures

Pigs were subjected to 1 of 3 euthanasia treatments: CO_2_ after receiving 0.085 mL saline i.m. (CO_2_), CO_2_ after receiving a dose calculated at 0.2 mg/kg BW for a 4.25 kg piglet, i.m. (0.085 mL dose) of butorphanol (CO_2_B), or N_2_O (N_2_O, no injection received). Butorphanol is an analgesic commonly used in veterinary medicine. Butorphanol is a synthetic partial agonist-antagonist analgesic, acting on kappa- and mu-opioid receptors [15], with a potency 7 times greater than morphine. It reaches its maximum analgesic effect within 20 to 30 min. The treatment with butorphanol was included to determine if specific aversive behaviors (‘pain behaviors’) could be verified that have been reported in pigs when they are euthanized with CO_2_. Many of the behaviors reported as aversive/painful are confounded with the body’s natural response to obtain air. In contrast, a fourth treatment using butorphanol was not included for the N_2_O treatment because no such aversion has been reported in humans when it is used in the dental industry and pigs also do not find it aversive [6].

Farm staff collected the pigs directly from the sow and delivered them in carts in groups of 6 to 8, until a new group was needed. The pigs were kept in a cart in a group and more pigs were brought in such that two pigs always had the company of at least two other pigs. Pigs in the CO_2_B treatment received butorphanol 30 min prior to treatment. Pigs were euthanized in pairs. When possible, a male and female were placed into the chamber, but because there were more males, often, two males entered the chamber. Our previous experiment [12] used groups of 4, but because these pigs were larger, euthanizing in pairs provided more space to stand and walk in the chamber, which would not have been possible if 4 pigs were used. Two cameras (KPC-N502NUB, KT&C, Fairfield, NJ, USA) were positioned on two sides of the acrylic glass windows into the chamber and video was recorded using video management software (GeoVision Network Video Recorder GV-NVR, Taipei, Taiwan). The video was recorded to quantify their behavior (Table 1) and was later analyzed with a software program (Observer XT 11, Noldus, Wageningen, The Netherlands). Behaviors indicative of activity, loss of consciousness, and distress were recorded (Table 1) [12]. When that rhythmic gaping ceased the gas was turned off. After no further movement was detected, the pigs were then taken out of the chamber, and a lack of heartbeat confirmed that each pig was dead. The pig’s body weight and sex were then recorded.

### 2.3. Data Processing

The duration (the sum of time when expressing the behavior), latency (the difference between the start time and the time the event started), and event data from the behavioral software were totaled for each pig. Data for the two pigs were then averaged for each repetition prior to analyses. Vocalizations were divided by the total time, which ended as defined by the cessation of gaping. No pigs were observed to panic for any of the treatments; thus, no data were analyzed. In six instances, pigs did not paddle, and this occurred in either of the CO_2_ treatments; thus, the sample size was not always *n* = 9.

### 2.4. Statistical Analysis

Data were analyzed using a general linear mixed model (Proc Glimmix, SAS version 9.4., SAS Institute Inc., Cary, NC, USA) and was checked for normality and homogeneity of variance prior to analysis. The treatment of euthanasia gas was included as a fixed effect with repetition as a random effect. Means of significant effects were separated with a Tukey’s adjustment. Log transformations were performed on data not meeting the statistical assumptions: Number of paddle bouts, latency to last movement, total time duration, and number of escape attempts. Influential outliers were detected with a Cook’s D test. One outlier was removed from the number of squeals. Data are presented as arithmetic means and standard error of means (means ± SE).

## 3. Results

The average body weight of pigs (*p* = 0.3306) and the average duration of gas exposure (*p* = 0.8223) among the three treatments did not differ (Table 2).

### 3.1. Duration and Latencies of Behaviors

Pigs spent a similar amount of time lying, being inactive, and in locomotion in all treatments (*p* > 0.05; Table 3). Pigs in the CO_2_B treatment spent the shortest time standing, with pigs in CO_2_ being intermediate and pigs in N_2_O being the longest (*p* < 0.001; Table 3). While the total duration of heavy breathing was shortest in the CO_2_B treatment (*p* < 0.03), the duration of open mouth breathing was shortest in the N_2_O treatment.

**Figure 1:**
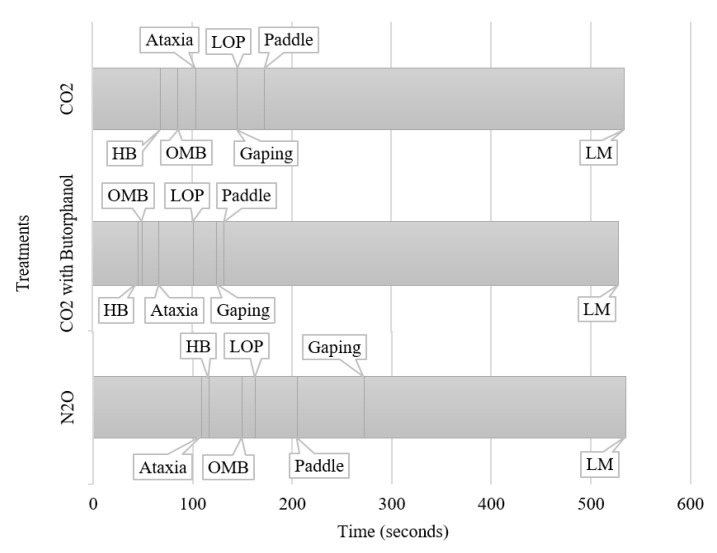
The sequence of behaviors (plotted from data in Table 3), showing latencies and durations, for pigs in each treatment: CO_2_, CO_2_ with butorphanol, and N_2_O. Using the means of each latency, a timeline was created to highlight the difference in the order in which the behaviors occurred. HB = heavy breathing, OMB = open-mouth breathing, LOP = loss of posture, LM = last movement. Postural behaviors standing and lying were mutually exclusive; and the activity behaviors paddling, inactivity, and locomotion were mutually exclusive.

Latency to heavy breathing (*p* = 0.0018) and gaping (*p* < 0.001) were shorter for CO_2_ and CO_2_B pigs compared to N_2_O pigs. Latency to open-mouth breathing (*p* < 0.001) and paddling (*p* < 0.001) time was the shortest in the CO_2_B treatment, was intermediate in the CO_2_ treatment, and was longest in the N_2_O treatment (Table 3). Latency to ataxia (*p* = 0.0104) and loss of posture (*p* < 0.0001) were similar for CO_2_ and N_2_O pigs but shorter for CO_2_B pigs (Table 3). Figure 1 provides a graphical depiction of the sequence of behaviors, showing the latencies and durations for each behavior for each treatment.

### 3.2. Behavioral Events

The rate of righting attempts and grunts did not differ significantly between treatments (*p* > 0.05; Table 4). Pigs performed more escape attempts in the CO_2_B treatment than pigs in the CO_2_ or N_2_O treatments (*p* = 0.0004). Pigs in the N_2_O treatment performed more paddle bouts (*p* = 0.0002) and squeals (*p* = 0.0016) than those in the CO_2_ or CO_2_B treatments (Table 4).

## 4. Discussion

The results partially support our first hypothesis that using N_2_O at a greater flow rate than our previous study [12] achieved a comparable time to loss of posture as CO_2_, and it reduced escape attempts by the pigs. Nonetheless, pigs exposed to N_2_O performed a higher number of squeals per minute than pigs exposed to CO_2_. However, the results do not support our second hypothesis that giving butorphanol to the pigs prior to exposure of CO_2_ would help us to elucidate behaviors indicative of pain because pigs given butorphanol actually made more escape attempts and the same number of squeals as CO_2_ pigs.

The onset of loss of posture and ataxia indicates that the animal starts to lose consciousness [5,16]. However, Smith et al. [12] noted that animals could make righting attempts after the loss of posture showing that they are not unconscious; thus, it is not clear for how long after losing posture the animal can feel pain or distress. For a euthanasia method to be more humane, death should occur as soon as possible with minimal pain and distress. Therefore, shorter latency to ataxia and loss of posture is considered as an indicator of the humaneness of the method. Nevertheless, if the animal is not feeling pain or distress, then the euthanasia method would be considered humane even if the latency to loss of posture was longer. Thus, the alleviation of pain and distress is more important than the length of the euthanasia process [17].

The latency to loss of posture and ataxia did not differ between pigs in the CO_2_ and N_2_O treatments. In our previous study [12], we used N_2_O with a 25% replacement rate and pigs had a longer latency to loss of posture. By increasing the flow rate to 50%, we were able to induce loss of posture as quickly as when CO_2_ is used at a 25% replacement rate. These data support our hypothesis that an increased flow rate of N_2_O could induce unconsciousness as quickly as CO_2_ at a 25% replacement rate.

Breathlessness is considered a very aversive experience, especially when exposed to hypercapnia [13,18]. Therefore, we consider heavy breathing and open-mouth breathing to be distressful behaviors, which are associated with compromised welfare [5,13]. Open-mouth breathing occurs just before the loss of posture when pigs were euthanized with CO_2_ [14,19,20]. The latency to heavy breathing and open-mouth breathing started earlier in pigs exposed to CO_2_ compared to N_2_O. Similarly, pigs exposed to CO_2_ experienced this respiratory distress (HB and OMB) for a longer duration of time than pigs exposed to Ar [20]. In our study, we had similar results in terms of latency to heavy breathing between pigs of CO_2_ and CO_2_B treatments; pigs in CO_2_ had a shorter latency to respiratory distress compared to pigs in N_2_O. However, while there was no difference in the duration of heavy breathing between pigs in CO_2_ and N_2_O, pigs in N_2_O had a shorter duration of open-mouth breathing compared to pigs in CO_2_. Because heavy breathing and open-mouth breathing are indicative of distress [13], limiting the duration of these behaviors increases the humaneness of the procedure. Thus, even though latency to heavy breathing and open-mouth breathing was shorter in the CO_2_ treatments, N_2_O pigs spent less time open-mouth breathing. We contend that heavy breathing is less stressful than open-mouth breathing because the latter is an exaggerated form of the former and always follows it on the time sequence, thus it is more intense and distressful. The observed results are likely due to CO_2_’s main action by hypercapnia, stimulating the acid-sensing ion channels in the medulla to cause an increase in the breathing rate and depth [21]. In contrast, N_2_O administration does not stimulate these receptors but rather acts by hypoxia, and gases that cause these effects through hypoxia rather than hypercapnia appear to minimize distress in pigs [6].

Escape attempts are considered evidence of stress or aversion in pigs during euthanasia [14,22]. In our study, pigs in the CO_2_ treatment had 14 times more escape attempts than pigs in the N_2_O treatment. On the other hand, pigs in N_2_O had 3.2 times more squeals than pigs in the CO_2_ treatment. This result is similar to experiment 2 in our previous research [12]; but in contrast to experiment 1 in the same study, we found no difference in squealing. The difference in methods between the two experiments was that experiment 1 euthanized only one pig at a time whereas in the experiment 2 groups of four to six pigs were euthanized together. We also found that pigs exposed to N_2_O in different combinations with other gases, or by itself did not squeal (6,11) upon exposure. Each study differed slightly, thus variables, such as the number of pigs and gas mixtures, may influence whether pigs squeal or not. Da Silva Cordeiro et al. [23] noted that animals in pain emit longer vocalizations and that it is critical to determine the duration, frequency, and amplitude to accurately determine if the vocalization is due to pain or another stressor. Consequently, we cannot be certain if the pig squeals recorded in this study were due to pain or distress (isolation from their dam and siblings) or if indeed N_2_O caused more pain than CO_2_. Given that N_2_O is commonly used in humans for its analgesic property with no known painful response, it seems unlikely that this is a stress response, but this finding is intriguing.

There was no difference between the CO_2_ treatments and the N_2_O treatment in the number of righting attempts. This observation in conjunction with the fact that CO_2_ pigs and N_2_O pigs lost posture at similar latencies suggests that they commenced unconsciousness at similar times. Pigs in both the CO_2_ and N_2_O treatment made approximately two righting attempts after losing posture. Thus, they quickly lost the ability to attempt to regain a standing posture.

Paddling is uncoordinated clonic convulsions that start after the onset of loss of consciousness and when the central nervous system has lost control of the brain stem and spinal cord [24]. Similarly, gaping is deep, rhythmic, and forceful breathing movements of the jaw and paired movements of the chest, which indicates respiratory arrest and is also coordinated by the brain stem [25]. Both behaviors (gaping and paddling) are indications that the brain is becoming ‘brain dead’ [25]. Latency to gaping and paddling was shorter in pigs of the CO_2_ treatments than those in the N_2_O treatment. Thus, although N_2_O pigs and CO_2_ pigs lost posture at similar times, it seems that the CO_2_ pigs became brain dead more quickly. In our previous work, we employed a lower flow rate of N_2_O, and CO_2_ pigs also had a quicker latency to paddling and gaping behaviors compared to N_2_O pigs [12]. Therefore, the quicker flow rate could shorten the latency to the onset of loss of consciousness but not to brain death. Possibly, a new protocol using a two-step procedure in which N_2_O is administered at a 50% replacement rate and then CO_2_ is delivered could be efficacious.

While pigs in the CO_2_ treatments showed HB, OMB, and then ataxia in consecutive order, pigs in the N_2_O treatment showed ataxia first, and then showed HB and OMB consecutively. This is an important indication that the N_2_O pigs are starting to lose central nervous system control prior to experiencing distress due to air hunger, possibly making the sensation of HB and OMB less distressful, although further research is needed to confirm this hypothesis.

Butorphanol was used prior to exposure to CO_2_ to determine if we could identify behaviors indicative of pain, with possible candidates to include squeals and escape attempts. If painful behaviors could be identified they would be useful in future experiments when comparing alternative gas euthanasia methods. Compared to the CO_2_ pigs, latency to ataxia, loss of posture, and paddling were shorter for CO_2_B pigs. These results are likely due to direct suppressive effects on the central nervous system and less likely informative about the pain pigs may have experienced. Thus, butorphanol had a positive effect on shortening the time to loss of consciousness and brain death, but it remains unfeasible for use as an on-farm euthanasia method because it is not approved for use in swine and would require the presence of a veterinarian. There was no difference in latency to HB between pigs in CO_2_ and CO_2_B treatments, but latency to OMB was shorter for CO_2_B pigs. More importantly, even though there was no difference in the duration of OMB between the CO_2_ and CO_2_B treatment, pigs in the CO_2_B treatment had a shorter duration of HB compared to the CO_2_ treatment. Butorphanol is known to have side effects, which can include respiratory depression, but this did not seem to be the case in this study since pigs in both treatments had the same duration of open-mouth breathing. The shorter duration of HB is likely due to butorphanol depressing the central nervous system while the pigs progress to loss of posture and paddling more quickly.

The number of righting attempts and squeals between the CO_2_ treatments did not differ. This implies that either these behaviors are not associated with pain or that the dosage of the butorphanol was insufficient to decrease behaviors that may be indicative of pain, such as squeals. Alternatively, it could be that the stress of breathlessness is so severe that it over-rides the sensation of pain. It is unlikely that the dose was not sufficient though, as this is the dose that is recommended for swine and proven to be a successful analgesic [26]. Similarly, the other possibility could be that the drug had not reached its full analgesic potential, but again, proven pharmacologic data suggest this would not be the problem either, with the maximal effect starting around 30 min. Interestingly, the pigs in the CO_2_B treatment made more escape attempts. It is unclear why this occurred, but possibly, if the pigs in CO_2_B were not suffering as much from HB and OMB, then they could focus on escaping which typically occurs when pigs are separated from their dam at weaning and put into a novel environment. The sequences of HB, OMB, ataxia, loss of posture, gaping, and paddling were similar for pigs in the CO_2_ and CO_2_B treatment, although typically, the CO_2_B pigs entered these stages sooner than CO_2_ pigs, indicating that the drug was affecting the central nervous system. Unfortunately, the compilation of these results, comparing using CO_2_ with or without butorphanol, did not allow us to clearly identify behaviors indicative of pain when pigs are exposed to CO_2_.

## 5. Conclusions

Weaned pigs euthanized with CO_2_ or N_2_O gases lost posture and became ataxic after a similar length of time, suggesting a similar efficacy. Pigs exposed to N_2_O displayed less OMB, which is a behavior indicative of distress but also squealed more often, which suggests greater distress or pain.

Pigs administered the butorphanol analgesic prior to exposure of CO_2_ made more escape attempts, a similar number of squeals, and less heavy breathing but similar OMB. Whether HB relates to pain or merely to respiratory depression should be further explored.

Overall, these results show that N_2_O can be as effective as CO_2_, and may be more humane although further research is needed to dissociate whether behaviors, such as squeals, HB, and OMB, indicate pain or distress.

## Figures and Tables

**Table 1:** Behavioral states and events recorded during euthanasia. Interruptions shorter than 3 s were considered the same bout of behavior. Behavioral recording started when the gas was turned on.

Category	Behavior	Description
Duration	Stand	Up on 4 legs
	Lying	Lying down with side or sternum contact with the floor
	Locomotion	Any movement more than 2 steps, walk or run
	Inactive	Immobile, not doing any particular behavior
Latency	Ataxic	Lack of muscles coordination in basic movements, loss of balance on one or more feet
	Loss of Posture	Lying on ground and does not get back up
	Heaving Breathing	Forceful and quick repetition of flank movements, mouth closed
	Open Mouth Breathing	Jaw held open with mouth open
	Gaping	Deep forceful breath, rhythmic movements of the chest with mouth open
	Last Movement	Clinically dead, stops gaping, end of experiment time
	Paddle Bout	Rhythmic movements of one or more legs while lying
	Panic/Convulsing Bout	Erratic, uncontrolled movements including flips, flops, thrashing before loss of posture
Events	Righting Response *	Unsuccessful effort to right up on 4 legs
	Escape Attempt *	Rear on its hind legs, jump, or scratch with front legs against the walls or the floor
	Squeal *	High-pitched vocalization; extended sound of high amplitude and frequency
	Grunt *	Low-pitched vocalization; sound of low to medium amplitude.

* Behaviors recorded as events due to their brief nature, rather than as states.

**Table 2:** Data collected (mean ± SE) on individual pigs for the three treatments.

Variable ^1^	Carbon Dioxide	Carbon Dioxide with Butorphanol	Nitrous Oxide	*p*-Value
Avg. Weight (kg)	4.47 ± 0.38	4.63 ± 0.0.42	3.97 ± 0.29	0.3306
Total duration (sec)	559.44 ± 24.47	544.11 ± 23.81	556.44 ± 21.31	0.8223

^1^ kg = kilogram, sec = second.

**Table 3:** Behavioral state results (mean ± SE) for multiple pairs of pigs subjected to the three treatments. Duration refers to the total elapsed time from onset to the cessation of the indicated variable. For the latency category, the numbers in parentheses indicate how many animals out of 18 in each treatment performed that specific behavior listed under column 2, “Variable”.

Category	Variable (s) ^1^	Carbon Dioxide	Carbon Dioxide with Butorphanol	Nitrous Oxide	*p*-Value
Duration	Standing	123.20 ± 10.83 ^b^	84.35 ± 3.35 ^c^	144.55 ± 5.75 ^a^	<0.0001
	Lying	432.28 ± 26.72	446.97 ± 26.34	407.69 ± 23.21	0.4508
	Paddling	31.09 ± 7.51 ^ab^	15.91 ± 2.92 ^b^	43.35 ± 9.83 ^a^	0.0294
	Inactivity	423.58 ± 39.97	475.01 ± 26.06	487.80 ± 22.98	0.9253
	Locomotion	81.62 ± 25.26	55.56 ± 5.37	63.06 ± 8.06	0.4564
	Heavy Breathing	26.07 ± 10.63 ^a^	2.50 ± 1.73 ^b^	34.41 ± 9.77 ^a^	<0.03
	Open Mouth Breathing	49.47 ± 7.26 ^a^	59.35 ± 10.34 ^a^	19.30 ± 8.61 ^b^	<0.01
Latency	Heavy Breathing	68.37 ± 11.07 ^b^	45.96 ± 11.96 ^b^	116.42 ± 9.77 ^a^	0.0018
		(10)	(16)	(18)	
	Open Mouth Breathing	85.64 ± 11.58 ^b^	49.69 ± 5.66 ^c^	149.15 ± 19.02 ^a^	<0.0001
		(18)	(1)	(9)	
	Gaping	145.50 ± 12.84 ^b^	124.97 ± 7.56 ^b^	272.50 ± 9.07 ^a^	<0.0001
		(10)	(10)	(10)	
	Ataxia	103.87 ± 11.46 ^a^	66.41 ± 4.24 ^b^	108.99 ± 6.57 ^a^	0.0104
		(17)	(18)	(18)	
	Loss of Posture	144.99 ± 17.27 ^a^	101.19 ± 4.67 ^b^	162.64 ± 6.99 ^a^	<0.0001
		(18)	(18)	(18)	
	Paddling	172.49 ±12.78 ^b^	132.27 ± 5.96 ^c^	204.84 ± 6.22 ^a^	<0.0001
		(16)	(14)	(17)	

^1^ s = second; ^a,b,c^ Means within a row with unlike superscript letters differed (*p* < 0.05).

**Table 4:** Behavioral event results (mean ± SE) on individual pigs for the three treatments. The numbers in parentheses indicate how many animals out of 18 in each treatment performed that specific behavior listed under column 2, “Variable”.

Variable (Frequency) ^1^	Carbon Dioxide	Carbon Dioxide with Butorphanol	Nitrous Oxide	*p*-Value
Escape Attempts	4.67 ± 1.55 ^a^	16.44 ± 4.23 ^b^	0.3333 ± 0.24 ^a^	0.0004
	(14)	(16)	(4)	
Righting Attempts	2.78 ± 0.60	1.11 ± 0.45	2.33 ± 0.75	0.2212
	(16)	(12)	(14)	
Paddle Bouts	6.11 ± 1.09 ^b^	2.11 ± 0.39 ^b^	10.56 ± 1.46 ^a^	0.0002
	(16)	(14)	(17)	
Squeals/(min)	2.80 ± 1.17 ^b^	1.28 ± 0.55 ^b^	9.02 ± 1.62 ^a^	0.0016
	(18)	(18)	(18)	
Grunts/(min)	16.46 ± 2.04	12.29 ± 2.03	20.97 ± 5.22	0.2015
	(18)	(17)	(18)	

^1^ min = minute, ^a,b^ Means within a row with unlike superscripts differ (*p* < 0.05).

## References

[1] (2017). Food and Agriculture Organization of the United Nations, FAOSTAT Statistical Database. http://faostat.fao.

[2] USDA (2015). “Swine 2012 Part I: Baseline Reference of Swine Health and Management in the United States, 2012” USDA–APHIS–VS, CEAH. Fort Collins, CO #663.0814. https://www.aphis.usda.gov/animal_health/nahms/swine/downloads/swine2012/Swine2012_dr_PartI.pdf.

[3] American Veterinary Medical Association (2013). Suckling Pigs. AVMA Guidelines for the Euthanasia of Animals.

[4] American Association of Swine Veterinarians (2008). National Pork Board On-Farm Euthanasia of Swine Recommendations for the Producer.

[5] Raj A.B.M., Gregory N.G. (1995). Welfare implications of the gas stunning of pigs: 1. Determination of aversion to the initial inhalation of carbon dioxide or argon. Anim. Welf..

[6] Rault J.L., McMunn K.A., Marchant-Forde J.N., Lay D.C. (2013). Gas alternatives to carbon dioxide for euthanasia: A piglet perspective. J. Anim. Sci..

[7] Steiner A.R., Axiak Flammer S., Beausoleil N.J., Berg C., Bettschart-Wolfensberger R., García Pinillos R., Golledge H.D., Marahrens M., Meyer R., Schnitzer T. (2019). Humanely Ending the Life of Animals: Research Priorities to Identify Alternatives to Carbon Dioxide. Animals.

[8] Zhang C., Davies M.F., Guo T.Z., Maze M. (1999). The analgesic action of nitrous oxide is dependent on the release of norepinephrine in the dorsal horn of the spinal cord. Anesthesiol..

[9] Clark M., Brunick A. (2003). N_2_O and its Interaction with the Body. Handbook of Nitrous Oxide and Oxygen Sedation.

[10] Thomas A.A., Flecknell P.A., Golledge H.D. (2012). Combining nitrous oxide with carbon dioxide decreases the time to loss of consciousness during euthanasia in mice—refinement of animal welfare?. PLoS ONE.

[11] Rault J.L., Kells N., Johnson C., Dennis R., Sutherland M., Lay D.C. (2015). Nitrous oxide as a humane method for piglet euthanasia: Behavior and electroencephalography (EEG). Physiol. Behav..

[12] Smith R., Rault J.L., Gates R., Lay D.C. (2018). A two-step process of nitrous oxide before carbon dioxide for humanely euthanizing piglets: On-farm trials. Animals.

[13] Beausoleil N.J., Mellor D.J. (2015). Introducing breathlessness as a significant animal welfare issue. N. Z. Vet. J..

[14] Velarde A., Cruz J., Gispert M., Carrión D., Torre R.J., Diestre A., Manteca X. (2007). Aversion to carbon dioxide stunning in pigs: Effect of carbon dioxide concentration and halothane genotype. Anim. Welf..

[15] Commiskey S., Fan L.W., Ho K., Rockhold R.W. (2005). Butorphanol: Effects of a prototypical agonist-antagonist analgesic on κ-opioid receptors. J. Pharm. Sci..

[16] Verhoeven M.T.W., Gerritzen M.A., Hellebrekers L.J., Kemp B. (2015). Indicators used in livestock to assess unconsciousness after stunning: A review. Animal.

[17] Hawkins P., Playle L., Golledge H., Leach M., Banzett R., Coenen A., Cooper J., Danneman P., Flecknell P., Kirkden R. (2006). Newcastle Consensus Meeting on Carbon Dioxide Euthanasia of Laboratory Animals. https://www.nc3rs.org.uk/sites/default/files/documents/Events/First%20Newcastle%20consensus%20meeting%20report.pdf.

[18] Lansing R.W., Gracely R.H., Banzett R.B. (2009). The multiple dimensions of dyspnea: Review and hypotheses. Respir. Physiol. Neurobiol..

[19] Sadler L.J., Haden C.D., Wang C., Widowski T.M., Johnson A.K., Millman S.T. (2014). Effects of flow rate and gas mixture on the welfare of weaned and neonate pigs during gas euthanasia. J. Anim. Sci..

[20] Raj A.B.M., Gregory N.G. (1996). Welfare implications of the gas stunning of pigs: 2. Stress of induction of anaesthesia. Anim. Welf..

[21] Detweiler N.D., Vigil K.G., Resta T.C., Walker B.R., Jernigan N.L. (2018). Role of acid-sensing ion channels in hypoxia-and hypercapnia-induced ventilatory responses. PLoS ONE.

[22] Dalmau A., Rodriguez P., Llonch P., Velarde A. (2010). Stunning pigs with different gas mixtures: Aversion in pigs. Anim. Welf..

[23] Da Silva Cordeiro A.F., De Alencar Nääs I., Oliveira S.R.M., Violaro F., De Almeida A.C.M., Neves D.P. (2013). Understanding Vocalization Might Help to Assess Stressful Conditions in Piglets. Animals.

[24] Terlouw E.C., Bourguet C., Deiss V., Mallet C. (2015). Origins of movements following stunning and during bleeding in cattle. Meat Sci..

[25] Raj A.B.M. (1999). Behaviour of pigs exposed to mixtures of gases and the time required to stun and kill them: Welfare implications. Vet. Rec..

[26] Ko J.C.H., Williams B.L., McGrath C.J., Short C.E., Rogers E.R. (1996). Comparison of anesthetic effects of Telazol-xylazine-xylazine, Telazol-xylazine-butorphanol, and Telazol-xylazine-azaperone combinations in swine. Contemp. Top. Lab. Anim. Sci..

